# Persistence of Reverse Transcription-Polymerase Chain Reaction (RT-PCR) Positivity in COVID-19 Recovered Patients: A Call for Revised Hospital Discharge Criteria

**DOI:** 10.7759/cureus.9048

**Published:** 2020-07-07

**Authors:** Tahir Jameel, Mukhtiar Baig, Zohair J Gazzaz

**Affiliations:** 1 Internal Medicine, King Abdulaziz University, Jeddah, SAU; 2 Clinical Biochemistry, King Abdulaziz University, Jeddah, SAU

**Keywords:** covid-19, rt-pcr, antibodies

## Abstract

In the world scenario, the advent of COVID-19 has halted every aspect of life. It influenced every field of life, including the economy, and revealed the inadequacies in all nations' healthcare systems, from the most developed to the underdeveloped countries. There is a debate about the timing of antibodies production and detection during the disease. What is the significance of reverse transcription-polymerase chain reaction (RT-PCR) viral ribonucleic acid (RNA) in symptom resolving period? In the present manuscript, we have evaluated these points.

## Introduction and background

The COVID-19 pandemic has adversely affected the world scenario. It has influenced every area of life, caused the loss of precious lives, and altered social, mental, and psychological well-being and global gross domestic product (GDP) [1]. However, this pandemic also brought the opportunity to improve our healthcare facilities. The diagnosis of COVID-19 depends on clinical presentation and essential investigations. The gold standard test remains the reverse transcription-polymerase chain reaction (RT-PCR) assay for the detection of viral ribonucleic acid (RNA) in oropharynx or nasopharynx. We have assessed RT-PCR and antibodies assay utility in COVID-19 patients in this brief review.

We searched the National Library of Medicine (PubMed) by using the search term “RT-PCR Test in COVID-19 Patients,” and “Antibodies Test in COVID-19 Patients,” and found 90, and 51, results, respectively. We narrowed our search by removing duplicate articles and abstracts, We downloaded full-text articles and selected only those articles that showed post-discharge viral RNA positivity.

## Review

**Figure 1:**
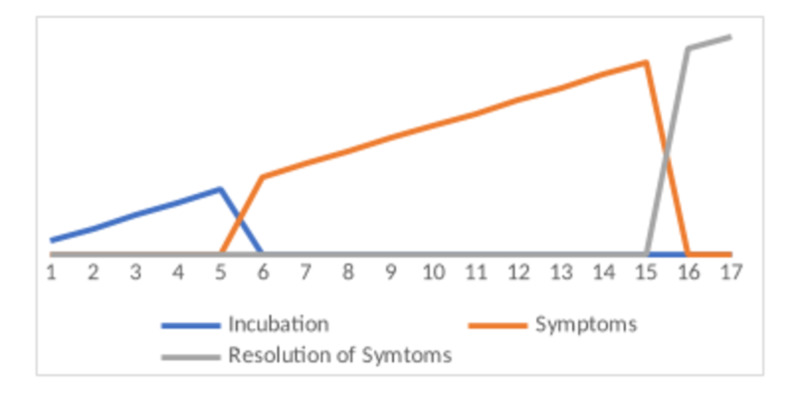
Clinical presentation among patients (number of days)

The emergence of COVID-19 has flipped the world upside down. Soon after its onset, the COVID-19 was isolated and recognized via real-time RT-PCR. Its incubation period is around three to nine days (Figure 1).

In around 44% of cases, the transmission of COVID-19 occurs before the symptomatic phase. About 18% of RT-PCR positive patients remain asymptomatic [2]. On recovery, symptoms disappear in almost ten days, but pharyngeal and oropharyngeal viral shedding is continued till around 8-19 days. The clearance of fecal shedding takes a longer time to resolve than the throat sample in almost 60%-70% of patients [2]. That’s why patients are advised to observe strict hygienic measures, especially after using the toilet. The hospital discharge criteria for COVID-19 patients upon recovery includes the absence of respiratory symptoms, afebrile for the last three days, radiological improvement of chest exudates (by X-ray or CT scan), and two upper respiratory tract samples negative for viral RNA in RT-PCR assay, collected at the interval of at least 24 hours. In the case of asymptomatic patients, the RT-PCR should be documented negative after 14 days [2].

Several researchers have reported that sometimes after satisfying all criteria of discharge from hospital or quarantine, patients continued to be RT-PCR positive for the next few days/weeks [3-7] (Table 1).

**Table 1:** Number of patients showing post-discharge viral RNA positivity *In patients receiving glucocorticoids, viral detection was prolonged RNA: ribonucleic acid.

Study	No of Patients	Post-discharge RT-PCR positivity for days	Clinical condition
Ling et al. [3]	11/66	15*	Stable
Lan et al.[4]	3/3	18	Stable
Xing et al.[5]	2/2	8	Stable
Zheng et al.[6]	20/20	7	Stable
Xu et al.[7]	8/8	7	Stable

There are certain possibilities, one being that RT-PCR can detect the presence of viral RNA irrespective of its virulence. During viral infections, the immunological system of the body produced specific antibodies against the infecting strain. The initial response is the production of immunoglobulin M (IgM), which can be detected up to three days of infection. After this, a highly specific IgG response can be observed. It plays a major role against the virus and is associated with signs of recovery. It also provides immune memory, and the person is resistant against the specific strain of the infecting virus [8]. In the majority of COVID-19 patients, the identification of IgM provides evidence of acute infection. In contrast, IgG specific to the virus strain protects the individual from succeeding reinfection and can be used in the form of immune serum therapy to serious patients. Over time IgM concentrations fall, and IgG remains high and exponential rise is seen in the case of reinfection [9].

It has been observed that viral RNA is present for a comparatively longer time in patients with comorbidities and were receiving glucocorticoids [3]. There is a need to revaluate the discharge criteria of COVID-19 patients as most of the patients have to wait because of RT-PCR finding. Instead, if we take the help of IgG antibody levels by sensitive techniques like enzyme-linked immunosorbent assay (ELISA), IgG's rising titers in a convalescent patient are diagnostic of recovery from the disease. WHO, in the latest newsletter “criteria for releasing COVID-19 patients from insolation," advised not to keep on waiting for RT-PCR to become negative; instead, the decision should be based on clinical and essential laboratory investigation like the presence of neutralizing antibodies. As in the presence of neutralizing antibodies, a non-symptomatic person cannot transmit the disease to others [10]. The number of cases of COVID-19 has increased so much that now the previously laid criteria of quarantine and admission in the hospital are being revised the world over because of the scarcity of resources as compared to the number of patients. 

## Conclusions

When the COVID-19 patient recovers, the discharge criteria from the hospital are meticulous, and the patient may wait a long time to be released from isolation because of the positive RT-PCR assay. Alternatively, if we use sensitive techniques such as ELIZA to measure IgG antibody levels, the rising IgG titers (neutralizing antibodies) in a convalescent patient diagnose the disease's recovery. The doctor-patient relationship is centered around the well-being and safety of our patients. At the same time, we should make sure that our patients do not create safety concerns for others. We hope that the subsequent research reports would add significant knowledge to the existing literature concerning patient care and safety.
